# Associations Between Air Pollution Exposure and Daily Pediatric Outpatient Visits for Dry Eye Disease: A Time-Series Study in Shenzhen, China

**DOI:** 10.3389/ijph.2021.1604235

**Published:** 2021-08-20

**Authors:** Jingfeng Mu, Dan Zeng, Jingjie Fan, Meizhou Liu, Shuyuan Yu, Wanfu Ding, Shaochong Zhang

**Affiliations:** ^1^ Shenzhen Eye Institute, Shenzhen Eye Hospital, Jinan University, Shenzhen, China; ^2^ School of Ophthalmology, Optometry, Shenzhen Eye Hospital, Shenzhen University, Shenzhen, China; ^3^ Shenzhen Maternal and Child Health Care Hospital, Shenzhen, China; ^4^ Shenzhen Center for Disease Control and Prevention, Shenzhen, China

**Keywords:** children, air pollution, generalized additive model, dry eye disease, Shenzhen

## Abstract

**Objectives:** This study aimed to investigate the associations between air pollution exposure and pediatric outpatient visits for dry eye disease (DED) in Shenzhen, China.

**Methods:** Generalized additive models were utilized to explore the acute effects of air pollution exposure on pediatric outpatient visits for DED.

**Results:** Single-day lag exposures to NO_2_, O_3_, PM_2.5_, and PM_10_ were associated with DED outpatient visits at lag days 0, 6, 4 and 2. Relative risks (RRs) for DED given a 10-μg/m^3^ increase in NO_2_, O_3_, PM_2.5_, and PM_10_ concentrations were 1.062[95% confidence interval (CI) 1.003, 1.123], 1.015(95% CI 1.001, 1.031), 1.052(95% CI 1.001, 1.115), and 1.038 (95% CI 1.002, 1.076), respectively. RR for DED given a 10-μg/m^3^ increase in NO_2_ over cumulative lag days 0–1 was 1.075 (95% CI 1.009, 1.147), and RR for DED given a 10-μg/m^3^ increase in PM_10_ over cumulative lag days 0–4 was 1.051 (95% CI 1.003, 1.102).

**Conclusion:** The observed associations between air pollution and outpatient visits for DED may provide evidence for policy makers to consider implementing measures to reduce the risk of DED owing to air pollution in China.

## Introduction

Dry eye disease (DED) is a disease of the ocular surface, characterized by distress, visual impairment, and tear film instability [1]. It is a multifactorial disease and is considered a disorder of the tear film caused by insufficient or excessive tear evaporation that damages the ocular surface and produces ocular discomfort [2]. Common DED symptoms include inability to produce tears, mucus overproduction, a burning sensation, itching, redness owing to light sensitivity, difficulty moving the eyelids, and pain [3]. Therefore, DED (with a current prevalence of 17–21% in China) represents an important and growing public health concern [4]. The prevalence of DED among ophthalmic outpatients in 32 cities in China was 61.57% as of 2013 [3].

Over the past decade, factors including a history of arthritis [5], prior ophthalmic surgery [6], age [7], sex [8], and contact lens usage [9] have been reported as major factors linked to DED risk. Frequent exposure of the ocular surface to air pollutants causes an increased vulnerability to these pollutants [10]. However, there have been few reports on the effects of air pollution on the ocular surface. In recent years, some studies have reported on associations between ocular toxicity and air pollution [11]. Hwang et al reported an association between high ozone levels and DED in South Korea [12]. Although air pollution is known to cause eye discomfort, irritation, redness, and blurred vision [13], it’s association with DED remains poorly understood [1].

The Pearl River Delta region, the site of Shenzhen city, is among the leading socioeconomic and urbanized areas in South China. However, rapid economic development and urbanization are linked with greater environmental concerns and demands on natural resources. Therefore, people in this region benefit from socioeconomic development but simultaneously experience the adverse effects of air pollution. According to epidemiological studies conducted in Shenzhen, cardiovascular and respiratory system diseases are associated with air pollution exposure [14, 15]. Air pollution has also been linked to infectious diseases [16], mortality [17], and low birth weight [18].

Air pollution is a global public health issue, and many epidemiological studies have revealed that air pollution accounts for numerous outpatient visits [19] and deaths [20]. However, most studies (especially those conducted in China) have focused on the effects of air pollution on the cardiopulmonary system, while has been rarely allocated to its effects on the eyes and ocular health. Children are most vulnerable to air pollution because their innate defenses to inhaled pollutants may be restricted owing to their developing lungs [21]. Therefore, air pollution poses a major threat to the health of children. As the first line of defense of the ocular surface, tear film is exposed to air and is particularly susceptible to air pollution [22, 23]. DED was once considered a rare disease in children because of insufficient reporting of eye discomfort and a lack of cooperation during eye examinations among this vulnerable population [24]. However, in recent years, the prevalence of DED in children has rapidly increased worldwide [25, 26], and more research is needed to evaluate this increase. Though additional research is necessary, the evaluation of associations between DED and environmental factors such as air pollution, altitude, and wind has already received increasing attention [27, 28]. For example, a study reported significant relationships between DED and air pollution in Hangzhou, China [1]. Nevertheless, few previous epidemiological studies have verified correlations between DED and air pollution in China, and no studies that have evaluated correlations between pediatric DED and air pollution in Shenzhen, China. To our knowledge, this study is the first effort to assess this correlation. Specifically, we conducted a time-series study using data from two hospitals to explore correlations between daily pediatric outpatient visits for DED and air pollution levels in Shenzhen, China. This study provides a basis for interpreting ocular toxicity associated with air pollution exposure.

## Methods

### Study Area

The present study was conducted in Shenzhen, China, a city located in the south of Guangdong Province, comprises 10 districts covering an area of 1997.47 km^2^, and had a population of approximately 13.4 million as of 2019. During the study period, an estimated 2,090,000 children between 0 and 18 years of age were living in Shenzhen, representing 15.6% of the total population. Few industrial pollution sources are located in the city, and vehicular emission is the main air pollution source. Shenzhen experiences a typical subtropical climate with an annual average temperature of 24°C.

### Study Population

We conducted this retrospective study to examine the associations between air pollution and pediatric DED. Data for outpatient visits at two hospitals (designated as Hospital A and Hospital B) occurring between January 1, 2018 and December 31, 2019 were obtained from their respective information systems. Hospital A is the largest ophthalmology clinic in Shenzhen with 200 beds, 458,000 annual outpatient visits, and 22,000 inpatients reported in 2019 [29]. Hospital B is the largest children’s medical center in Shenzhen with 1,292 beds, 2.5 million annual outpatient visits, and 80,000 inpatients reported in 2019 [29]. Hospital B serves 50.0% of the child patients in Shenzhen [29]. Hospital A and Hospital B serve 68.3% of the patients with eye diseases in Shenzhen (57.8% for Hospital A and 10.5% for Hospital B) [29]. Patients younger than 18 years visiting the Hospital A or Hospital B for DED were included in this study. Diagnosis of DED was performed in Hospital A and Hospital B according to the International Dry Eye WorkShop [30]. The International Classification of Diseases, 10th Revision (ICD–10) was used to define the outcomes and H11.103 represented DED. The number of patients as well as the visit dates were obtained and abstracted from the respective information systems. The present study was limited to patients’ first hospital visit. Patients who were residents in Shenzhen for less than 6 months during the study period were excluded from the current investigation to reduce potential biases.

### Outdoor Air Pollutants and Meteorological Data

Publicly available outdoor air pollution data for sulfur dioxide (SO_2_), nitrogen dioxide (NO_2_), particulate matter <10 μm in diameter (PM_10_), carbon monoxide (CO), particulate matter <2.5 μm in diameter (PM_2.5_) and ozone (O_3_) between January 1, 2018 and December 31, 2019 were retrieved from the Shenzhen Municipal Ecological Environmental Bureau (http://meeb.sz.gov.cn/). There were 11 fixed-site air quality-monitoring stations equipped with automatic exposure assessment machines across Shenzhen that monitored daily concentrations of air pollutants during the study period. PM_10_ (daily 24-h average), NO_2_ (daily 24-h average), PM_2.5_ (daily 24-h average), SO_2_ (daily 24-h average), CO (daily 24-h average), and O_3_ (maximum daily 8-h average) concentrations were tracked at each monitoring station. The monitoring stations were located far from obvious emission sources to ensure the representativeness of the general pollution levels [31]. The mean concentrations of PM_10_, NO_2_, PM_2.5_, SO_2_, CO, and O_3_ measured at these 11 monitoring stations represent the exposure levels of PM_10_, NO_2_, PM_2.5_, SO_2_, CO, and O_3_ in Shenzhen in this study. There was no missing air pollution data at any of the monitoring stations during the study period.

Publicly available meteorological data (principally daily temperature and relative humidity) were collected from the Meteorological Bureau of the Shenzhen Municipality (http://weather.sz.gov.cn/) from January 1, 2018 to December 31, 2019. No meteorological information was missing during the study period.

### Statistical Analysis

Generalized additive models (GAM) are widely used to assess the adverse effects of air pollution on health. GAM can not only fit independent variables that are linearly related to dependent variables but also fit independent variables that are nonlinearly related to dependent variables. It is advantageous to explore the essential relationships between dependent and independent variables. In this study, GAM were employed to assess the effects of ambient air pollution on DED outpatient visits. To control for confounding effects, we assessed the effects of relative humidity, temperature, day of the week (DOW), time trends, and holidays on DED occurrence in children. Categorical indicator variables were used to control for public holidays and DOW, while penalized smoothing splines were implemented to adjust for time trends in outpatient visits [32]. According to Hwang et al., temperature and relative humidity can affect tear stability, tear evaporation, and the thickness of the lipid layer [12], while Galor et al. indicated that meteorological factors such as relative humidity and temperature may affect the risk of DED [28]. Therefore, it was necessary to include temperature and relative humidity in the GAM. The main model is described as follows:

$$\log\left[E\left(Y_{i}\right)\right]=\sum_{j=1}^{m}f_{j}x_{j}+\sum_{i=1}^{n}\beta_{i}x_{i}+as.factor\left(DOW\right)+as.factor\left(H\right)+\alpha$$

where *E*(*Y*_*i*_) represents the expected number of DED outpatients on day i, *f*_*j*_ represents a natural cubic regression smooth function, *x*_*j*_ stands for variables that are nonlinearly associated with dependent variables such as temperature, time, and relative humidity, *x*_*i*_ denotes the concentration of air pollutants on day i, *β*_*i*_ represents the log-related DED outpatient rate associated with increased air pollutant concentrations, *DOW* represents the day of the week, and *H* represents public holidays.

We estimated potentially delayed associations to validate air pollution lag patterns. Up to a 6-days lag was incorporated into models for non-cumulative and cumulative exposures. Non-cumulative effects are defined as exposure effects for lag day 0 to lag day n, while cumulative effects are the mean concentration effects for lag days 0–n. According to previous studies, the lag effects of air pollutants are the strongest within 7 days [33, 34].

**FIGURE 1:**
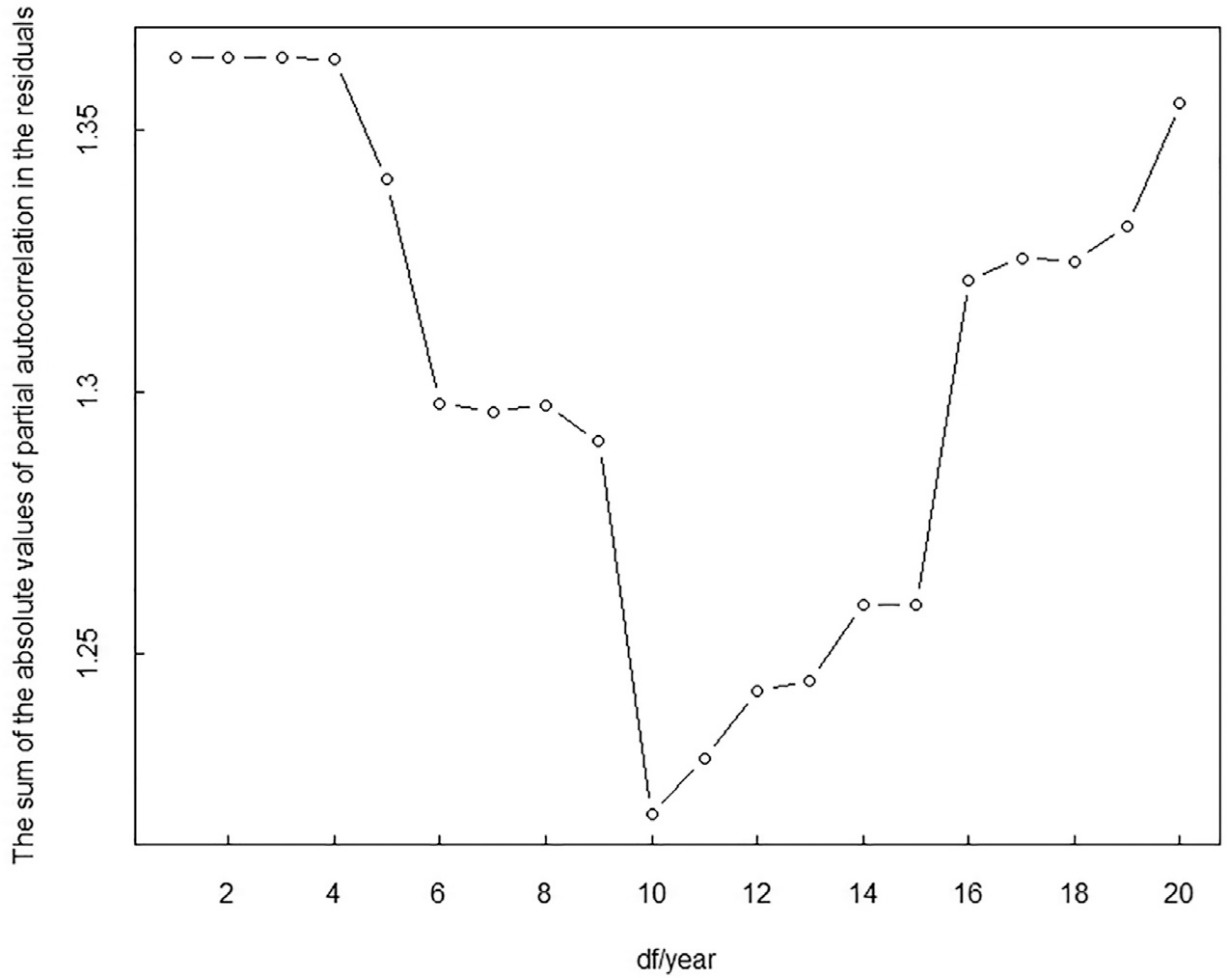
The sum of the absolute values for partial autocorrelation in the residuals of models with different degrees of freedom of time from January 1, 2018 to December 31, 2019 in Shenzhen, China (Shenzhen, 2018-2019).

The smoothing degree of GAM increases with decreases in degrees of freedom (df), but goodness of fit decreases simultaneously. Conversely, the smoothing degree of GAM decreases with increases in df, but goodness of fit increases simultaneously. Therefore, the selection of df plays a critical role in the construction of the optimal model. The df of time was selected based on a strategy to minimize autocorrelation in the residuals [35, 36]. As shown in Figure 1, the smoothing function of time involving 10 df/yr was used as a control for long-term trends. According to previous studies, the natural smoothing functions of temperature (associated with six df) and relative humidity (associated with three df) were utilized as controls for the effects of meteorological factors [37]. Akaike’s Information Criterion was used to select the df of temperature and relative humidity in the present study. After setting controls for the effects of DOW, holidays, long-term trends, and meteorological factors, the GAM was utilized to analyze the exposure–response relationships between daily outpatient visits for pediatric DED and air pollution levels. Relative risks (RRs) and associated 95% confidence intervals (CIs) were expressed as changes in daily DED outpatient visits per 10-μg/m^3^ increase in SO_2_, O_3_, NO_2_, PM_2.5_, and PM_10_ concentrations, and per 1-mg/m^3^ increase in CO concentrations. Single-day lag effect models of single-pollutants and cumulative effect models of single-pollutants were employed to evaluate associations between air pollution levels and pediatric DED. The results are presented as changes in daily DED admissions with increased air pollutant levels on different lag days.

We conducted Kolmogorov–Smirnov tests to verify the normality of the daily number of outpatient, the concentration of air pollutants, and meteorological data, with the results showing non-Gaussian distributions for all data. Spearman’s correlation analysis was conducted to evaluate the relationships between the parameters studied. In this study, *p*-values < 0.05 were considered to indicate statistical significance. Statistical analysis was performed using the “mgcv” package in version 3.6.3 of R software [38]. This study was approved by the Institutional Review Board of Hospital A (approval number: L-2020-002), with approval extended to Hospital B, and followed the principles of the Declaration of Helsinki. Informed consent from patients was not required because the data included in this study was anonymous and obtained from the information systems of the hospitals.

## Results

### Characteristics of Outpatients Reporting DED

**FIGURE 2:**
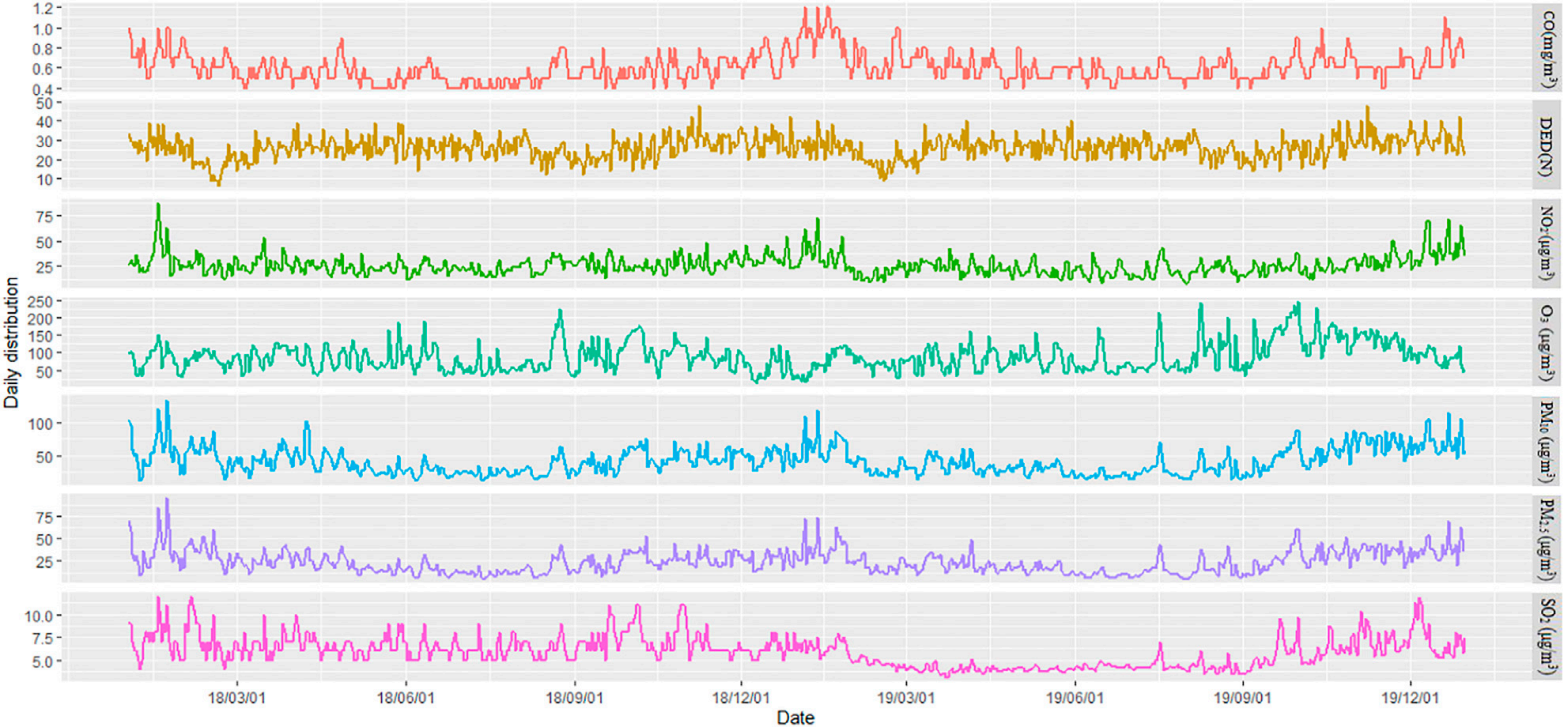
Time series of the daily number of outpatients with dry eye disease as well as air pollutant levels from January 1, 2018 to December 31, 2019 in Shenzhen, China (Shenzhen, 2018-2019).

Table 1 presents the descriptive statistics of the daily number of outpatient visits for pediatric DED. In Figure 2, the time series for the daily number of outpatients with DED in Shenzhen during the study period, shows values ranging from 1 to 52. The lowest number of outpatients was observed in June, while the highest was observed in November. In total, 19,170 children who visited either Hospital A or Hospital B during the study period were diagnosed with DED. 16,977 children and 2,193 children visited Hospital A and Hospital B owing to DED, respectively. The characteristics of outpatients with DED in the present study are summarized in Table 1, revealing a mean daily number of 26.26 cases occurring during the study period.

**TABLE 1:** Descriptive statistics for the daily number of outpatients with dry eye disease (DED), daily concentrations of air pollutants, and meteorological data from January 1, 2018 to December 31, 2019 in Shenzhen, China (Shenzhen, 2018-2019).

Variables	Unit	Days	Mean	SD	Min	*P*_25_	*P*_50_	*P*_75_	Max	Kolmogorov–Smirnov test	
										*Z* value	*p* value
Daily number of outpatients with DED	N	730	26.26	11.07	1	19	26	33	52	4.31	<0.001
Hospital A	N	730	23.52	10.33	1	17	23	29	47	3.07	<0.001
Hospital B	N	730	2.74	1.39	0	3	4	5	5	2.53	<0.001
Air pollutants											
PM_2.5_	µg/m^3^	730	24.09	12.59	5.0	14.0	22.0	32.0	95.0	1.81	0.003
PM_10_	µg/m^3^	730	41.09	20.23	11.0	25.0	37.0	54.0	134.0	2.28	<0.001
SO_2_	µg/m^3^	730	5.91	1.69	3.0	5.0	6.0	7.0	12.0	4.80	<0.001
CO	mg/m^3^	730	0.61	0.14	0.40	0.50	0.60	0.70	1.20	4.95	<0.001
NO_2_	µg/m^3^	730	26.03	9.79	7.0	19.0	25.0	31.0	87.0	1.94	0.001
O_3_	µg/m^3^	730	88.15	40.82	14.0	57.0	82.0	111.0	245.0	1.78	0.003
Meteorological data											
Temperature	°C	730	23.40	5.41	6.6	20.0	24.4	27.8	30.8	2.03	0.001
Relative humidity	%	730	75.96	12.94	23.0	72.0	79.0	84.0	98.0	2.65	<0.001

SD, Min, *P*_25_, *P*_50_, *P*_75_, Max: standard deviation, minimum, 25th percentile, 50th percentile, 75th percentile, and maximum, respectively.

### Characteristics of Air Pollutants and Meteorological Factors

According to the descriptive statistics presented in Table 1 and the time series presented in Figure 2, the average [standard deviation (SD)] concentrations of NO_2_, CO, SO_2_, PM_2.5_, PM_10_, and O_3_ were 26.03 ± 9.79 μg/m^3^, 0.61 ± 0.14 mg/m^3^, 5.91 ± 1.69 μg/m^3^, 24.09 ± 12.59 μg/m^3^, 41.09 ± 20.23 μg/m^3^, and 88.15 ± 40.82 μg/m^3^, respectively. During the study period, the mean concentrations of air pollutants in Shenzhen were below the Level 2 of the ambient air quality standards in China [40 μg/m^3^ (NO_2_), 4 mg/m^3^ (CO), 60 μg/m^3^ (SO_2_), 35 μg/m^3^ (PM_2.5_), 70 μg/m^3^ (PM_10_), and 160 μg/m^3^ (O_3_)] [39]. The daily temperature ranged from 6.6 to 30.8°C, while daily humidity varied from 23.0 to 98.0%, with respective mean values of 23.4°C and 75.96%.

### Correction Analysis Between DED and Air Pollution

We report Spearman’s correlation analyses between morbidity data and air pollution. The daily number of outpatients diagnosed with DED showed positive correlations with SO_2_ (*r* = 0.553, *p* < 0.05), O_3_ (*r* = 0.538, *p* < 0.05), PM_10_ (*r* = 0.544, *p* < 0.05), PM_2.5_ (*r* = 0.605, *p* < 0.05) and CO levels (*r* = 0.066, *p* > 0.05). The daily number of outpatients diagnosed with DED showed negative correlations with NO_2_ (*r* = −0.002, *p* > 0.05), relative humidity (*r* = −0.525, *p* < 0.05), and temperature (*r* = −0.030, *p* > 0.05). Positive correlations were found between the concentrations of air pollutants (NO_2_, CO, SO_2_, PM_2.5_, PM_10_, and O_3_) in this study. For example, Spearman’s correlation coefficients were 0.974 between PM_2.5_ and PM_10_ (*p* < 0.05), 0.768 between PM_2.5_ and SO_2_ (*p* < 0.05), 0.796 between PM_10_ and SO_2_ (*p* < 0.05), and 0.623 between PM_10_ and NO_2_ (*p* < 0.05).

### DED Risk and Air Pollutant Exposure

Associations between the non-cumulative air pollution exposure and the daily outpatient visits involving DED are presented in Table 2. The results revealed statistically significant direct associations of O_3_ at lag day 6, PM_10_ at lag day 2, NO_2_ at lag day 0, and PM_2.5_ at lag day 4 with outpatient visits owing to DED (*p* < 0.05). The RR values associated with DED outpatient visits per 10-μg/m^3^ increase in O_3_ concentrations at lag day 6, PM_10_ at lag day 2, NO_2_ at lag day 0, and PM_2.5_ at lag day 4 were 1.015 (95% CI 1.001, 1.031), 1.038 (95% CI 1.002, 1.076), 1.062 (95% CI 1.003, 1.123), and 1.052 (95% CI 1.001, 1.115), respectively. The associations of other pollutants with DED were not statistically significant (*p* > 0.05). Associations between daily outpatient visits involving DED and cumulative air pollution exposure are presented in Table 2. According to these results, the concentrations of NO_2_ for lag days 0–1 and those of PM_10_ for lag days 0–4 showed a statistically significant direct association with outpatient visits owing to DED (*p* < 0.05). The RR values for DED outpatient visits associated with every 10-μg/m^3^ increase in NO_2_ concentrations for lag days 0–1 and every 10-μg/m^3^ increase in PM_10_ concentrations for lag days 0–4 were 1.075 (95% CI 1.009, 1.147) and 1.051 (95% CI 1.003, 1.102), respectively. We observed positive but statistically insignificant associations between the other pollutants and outpatient visits involving DED.

**TABLE 2:** Relative risks (RRs) with 95% confidence intervals (CIs) for dry eye disease outpatient visits for every 10-μg/m^3^ increase in NO_2_, O_3_, PM_10_, PM_2.5_, and SO_2_ exposures and for every 1-mg/m^3^ increase in CO from January 1, 2018 to December 31, 2019 in Shenzhen, China (Shenzhen, 2018-2019).

Lag	CO (1 mg/m^3^)	NO_2_ (10 μg/m^3^)	O_3_ (10 μg/m^3^)	PM_10_ (10 μg/m^3^)	PM_2.5_ (10 μg/m^3^)	SO_2_ (10 μg/m^3^)
Lag day 0	0.849 (0.551, 1.308)	**1.062 (1.003, 1.123)**	0.987 (0.973, 1.001)	1.014 (0.979, 1.049)	1.019 (0.965, 1.075)	1.032 (0.647, 1.647)
Lag day 1	0.757 (0.488, 1.176)	1.005 (0.946, 1.068)	0.992 (0.978, 1.007)	0.994 (0.960, 1.030)	0.986 (0.933, 1.041)	0.853 (0.531, 1.371)
Lag day 2	1.273 (0.810, 1.999)	1.016 (0.956, 1.081)	0.999 (0.984, 1.013)	**1.038 (1.002, 1.076)**	1.033 (0.977, 1.093)	1.212 (0.743, 1.978)
Lag day 3	1.079 (0.691, 1.684)	1.028 (0.968, 1.092)	1.001 (0.987, 1.016)	1.019 (0.984, 1.056)	1.026 (0.971, 1.084)	1.449 (0.889, 2.362)
Lag day 4	0.944 (0.602, 1.048)	1.020 (0.959, 1.085)	1.000 (0.986, 1.015)	1.025 (0.990, 1.063)	**1.052 (1.001, 1.115)**	0.755 (0.460, 1.242)
Lag day 5	0.709 (0.453, 1.110)	0.972 (0.912, 1.036)	0.995 (0.981, 1.010)	0.994 (0.957, 1.031)	0.977 (0.922, 1.036)	0.848 (0.510, 1.410)
Lag day 6	1.027 (0.652, 1.616)	1.023 (0.962, 1.087)	**1.015 (1.001, 1.031)**	1.016 (0.979, 1.054)	1.026 (0.969, 1.086)	1.189 (0.717, 1.972)
Lag days 0–1	0.768 (0.477, 1.239)	**1.075 (1.009, 1.147)**	0.988 (0.973, 1.003)	1.005 (0.967, 1.044)	1.003 (0.944, 1.066)	0.926 (0.556, 1.543)
Lag days 0–2	0.895 (0.533, 1.501)	1.038 (0.963, 1.120)	0.990 (0.973, 1.006)	1.019 (0.976, 1.063)	1.020 (0.953, 1.091)	1.016 (0.585, 1.766)
Lag days 0–3	0.948 (0.549, 1.635)	1.048 (0.967, 1.135)	0.991 (0.974, 1.008)	1.023 (0.978, 1.069)	1.026 (0.956, 1.102)	1.165 (0.654, 2.074)
Lag days 0–4	0.913 (0.507, 1.645)	1.049 (0.959, 1.147)	0.992 (0.975, 1.010)	**1.051 (1.003, 1.102)**	1.041 (0.963, 1.124)	1.031 (0.559, 1.901)
Lag days 0–5	0.802 (0.430, 1.494)	1.029 (0.934, 1.136)	0.995 (0.976, 1.014)	1.027 (0.975, 1.081)	1.029 (0.948, 1.118)	0.970 (0.509, 1.850)
Lag days 0–6	0.843 (0.443, 1.605)	1.041 (0.942, 1.152)	0.993 (0.974, 1.012)	1.030 (0.977, 1.086)	1.038 (0.953, 1.131)	1.030 (0.531, 1.997)

Bolded relative risks indicate statistical significance at the 0.05 level.

### Exposure–Response Association of Air Pollution and DED

**FIGURE 3:**
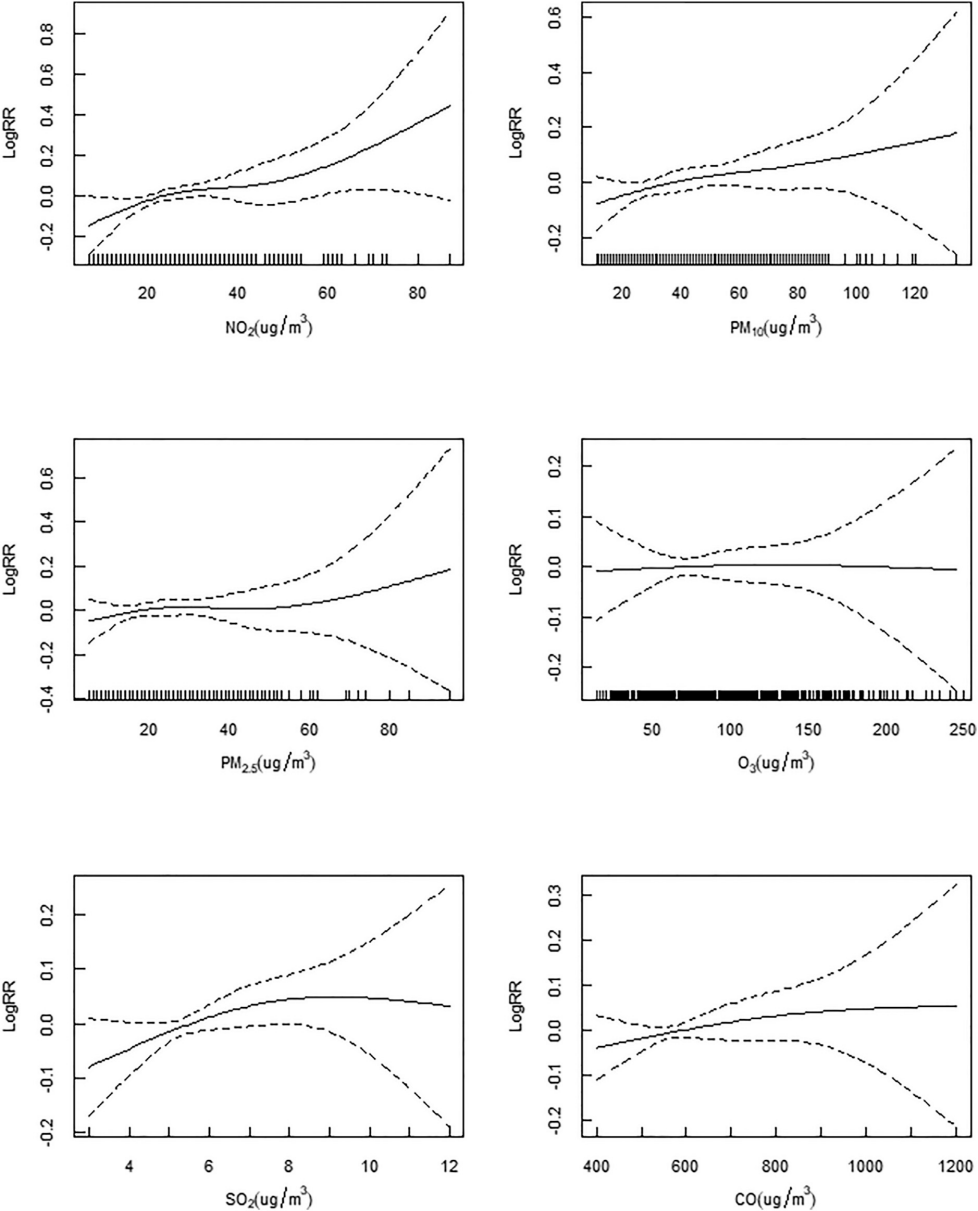
The exposure-response relationship curves of air pollutant concentrations with dry eye disease hospital outpatient visits from January 1, 2018 to December 31, 2019 in Shenzhen, China (Shenzhen, 2018-2019).

**FIGURE 4:**
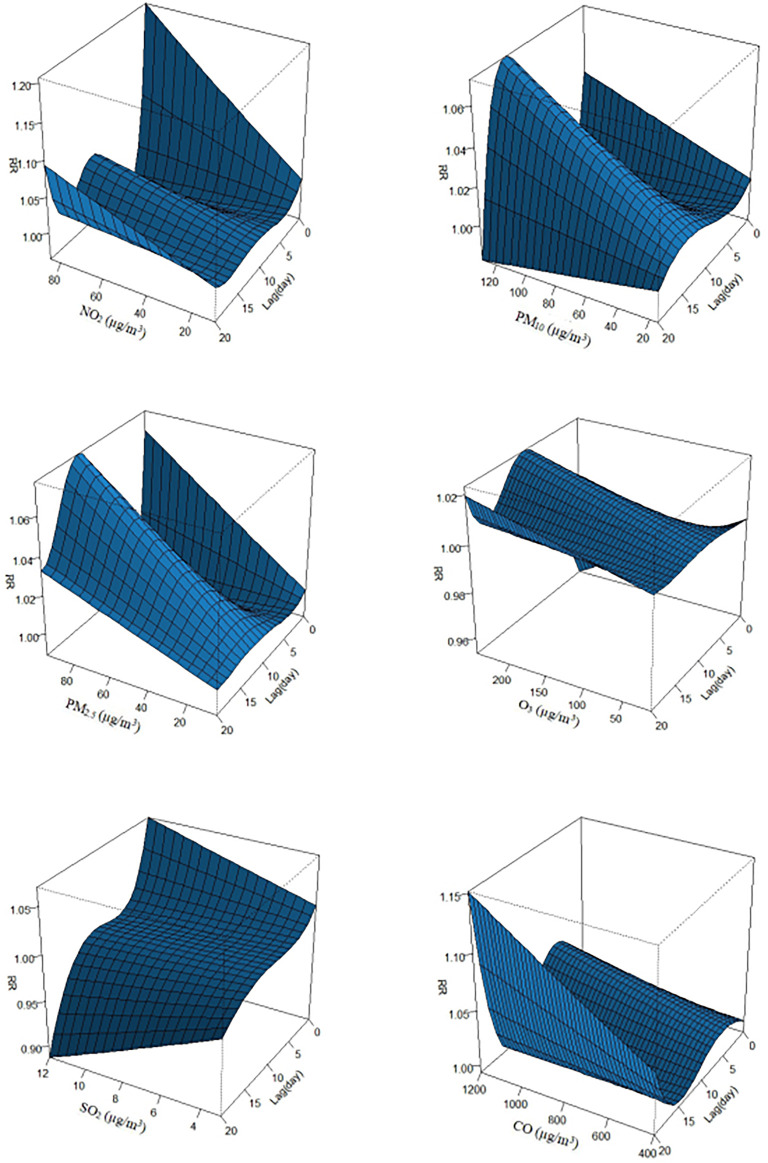
A three-dimensional plot of the association between air pollution exposure and dry eye disease hospital admissions over 20 lag days from January 1, 2018 to December 31, 2019 in Shenzhen, China (Shenzhen, 2018-2019).

The exposure–response associations between air pollution concentrations (O_3_ at lag day 6, PM_10_ at lag day 2, NO_2_ at lag day 0, PM_2.5_ at lag day 4, CO at lag day 2 and SO_2_ at lag day 3) and outpatient visits linked with DED are displayed in Figure 3. The exposure–response curves for DED caused by air pollution (NO_2_, PM_10_, PM_2.5_, O_3_, SO_2_, and CO) were almost linear. In general, the risk of DED increased with rising air pollutants concentrations, highlighting positive correlations between exposures to these pollutants and outpatient visits involving DED in our study. Even when the concentrations of air pollutants were much lower than the Level 2 of the ambient air quality standards in China [39], the risk of pediatric outpatient visits for DED continued to rise. The exposure-response associations and lag effects of pollutants with respect to DED were limited in terms of time dimensions and the exposure-response associations of different lag times were estimated in this study. Figure 4 shows a three-dimensional plot of the effects of air pollution exposure on 20 lag days of outpatient visits. This plot revealed that the association between air pollutant concentrations and the daily number of outpatient visits owing to DED was approximately linear without any thresholds. RRs of DED increased with increases in pollutant levels. Even when the concentrations of pollutants were very low, associations between pollutant exposures and DED risk were still statistically significant.

## Discussion

During the study period, statistically significant associations were found between air pollution levels and pediatric outpatient visits involving DED in Shenzhen, China. The exposure–response curves in our study were characterized by increasing trends. This study demonstrated that although concentrations of ambient air pollutants in Shenzhen were much lower than the regulations specified in federal air quality standard in China, air pollutants with low levels can contribute to DED in children. These results highlight the need for greater public health efforts to protect the population from the risk of DED, as well as other adverse effects of low-level ambient air pollution, and indicate that stricter control of air pollutants is required. Our findings will inform public health efforts and regulatory guidelines.

Children are commonly more exposed to air pollution than adults because they spend relatively longer periods of time outdoors [40]. During such periods, the ocular surface is constantly exposed to air and the eye is more susceptible to these pollutants than other organs [31]. Several previous studies reported correlations between air pollution and ocular toxicity [11]. Exposure to ambient air pollutants commonly causes eye discomfort, irritation, redness, blurred vision, and DED [13].

The results of the present study are consistent with those reported elsewhere. For example, Mo et al. conducted a study in Hangzhou, China and reported positive relationships between DED and air pollutants (including NO_2_ and PM_2.5_) [1]. Other studies reported increased risks of eye and adnexa diseases according to exposure to PM_10_ and SO_2_ [41], PM_2.5_, CO, and O_3_ [42]. During the study period, we observed linear relationships without any thresholds and lag effects between the air pollutants and DED among children. As the concentration of pollutants increased, the risk of DED increased concomitantly, which was in agreement with previous studies [43]. The effects of some air pollutants were inversely associated with their concentrations for some lagging periods (e.g., O_3_ at lag 0) (*p* > 0.05), which was likewise consistent with other study findings [44].

Several studies have suggested that ocular surface cell dysfunction is associated with DED. A study conducted in Hangzhou, China showed that PM_2.5_ inhibits the activity of ocular surface epithelial cells, thus damaging the ocular surface cells [45]. Air pollutants can alter the precorneal tear film (PTF), leading to ocular discomfort and subsequent DED diagnosis. A possible mechanism for this change involves disturbance of the PTF structure by PM_2.5_ [1]. The positive correlation between DED and PM_2.5_ in the present study is in agreement with previous studies [1]. To our knowledge, an established mechanism plausibly explaining the association between DED and air pollution remains elusive. However, this study demonstrated that air pollution is likely a risk factor for pediatric DED. Further laboratory and epidemiologic studies are needed to improve the current understanding of the relationship between DED and air pollution.

The present study is a novel attempt to investigate associations between air pollutants concentrations and outpatient visits for pediatric DED in Shenzhen, China. This study provides a unique opportunity to verify the exposure–response associations between DED and air pollution. The statistically significant effect of air pollution on pediatric DED revealed in this study highlights the need for adequate air pollution control. Further, GAM were performed to investigate associations between DED and air pollution exposure in the present study, demonstrating the suitability of this methodology for describing nonparametric smoothing functions and non-linear effects [46]. Simulations using the GAM are better than that of linearity-based models when non-linear effects are involved [47]. The GAM is more accurate in modeling and forecasting data exhibiting Poisson distributions [48, 49].

Our study had some limitations. First, because of its ecological design, our ability to derive causal inferences is limited. Therefore, the causality of the associations between DED and air pollution highlighted in the results may not be definitive. Second, in the present study, data on clinical visits were collected exclusively from Hospital A and Hospital B. Thus, the representativeness of the data relative to the population of Shenzhen is questionable, as is the overall generalizability of our study findings. Third, the concentrations of air pollutants measured in the air quality-monitoring stations may not accurately reflect individual exposures, which include mobility and residential components. Therefore, in the future, cohort studies to verify the effects of air pollution on ocular health as well as improved exposure assessment will be necessary.

In conclusion, the current study investigated relationships between outpatient visits for pediatric DED and air pollution levels in Shenzhen, China. Our results suggest that air pollution is a potential risk factor for DED, though the generalizability of our findings to other populations and to adults are unclear. NO_2_, O_3_, PM_10_, and PM_2.5_ concentrations exhibited direct associations with outpatient visits owing to DED. This study is the first effort to investigate the association between pediatric DED and air pollution in Shenzhen, China and one of few studies of this topic in general. Our study indicates that the government should consider stricter regulatory measures to protect the environment, public health, and the health of vulnerable populations, with the goal of reducing the health risks associated with air pollution in China.
